# Building sustainable primary healthcare systems: Lessons from Brazil and China

**DOI:** 10.1016/j.ghrp.2026.06.001

**Published:** 2026-06-07

**Authors:** Chen Chen, Jialong Tan, Luiz Andrade, Garibaldi Gurgel, Ivana Barreto, Jian Wang

**Affiliations:** a Department of Global Health, School of Public Health, Wuhan University, China; b Dong Fureng Institute of Economic and Social Development, Wuhan University, Wuhan, China; c Digital Health Research Group, Fiocruz Ceará, Brazil; d Federal University of Ceará, Brazil; e Fiocruz Pernambuco and Pernambuco School of Public Health Government, Brazil; f General Hospital of Fortaleza, Ceará State Health Department, Brazil

**Keywords:** China, Brazil, Primary Health Care, Digital Health, Family Health Strategy

## Abstract

Primary health care (PHC) is fundamental to achieving universal health coverage and health equity, yet building sustainable PHC systems remains a global challenge. This commentary compares the contrasting yet complementary experiences of Brazil and China along four analytical dimensions: community embeddedness, financial protection and sustainability, continuity of care and coordination across levels, digital support and its dual equity effect. Brazil’s Family Health Strategy (FHS), rooted in community-based multidisciplinary teams and a rights-based framework, has significantly reduced mortality from preventable conditions and expanded access for marginalized populations, but faces sustainability threats from underfunding and political instability. China, by contrast, has adopted a state-driven, technologically enabled approach, leveraging telemedicine, electronic records, and AI-enabled tools to scale up PHC. Despite impressive coverage and financial protection, China struggles with limited public trust in frontline providers, fragmented care continuity, and a notable dual pattern in digital health: telemedicine has helped narrow the rural-urban gap, while the gap between younger and older users has widened. Drawing on recent policy documents and empirical studies, we distinguish broadly transferable principles from resource-intensive pathways, offering low- and middle-income country (LMIC) policymakers an integrative framework that prioritizes human-centered relational care reinforced by context-appropriate digital tools, predictable financing, and strong referral linkages within an equity-focused PHC foundation.

## Background

PHC is widely acknowledged as the cornerstone of equitable, and people-centered health systems. As a foundational strategy for advancing health equity and achieving health-related Sustainable Development Goals (SDGs), PHC serves as the first point of contact between individuals and the health system, and a platform for delivering promotive, preventive, rehabilitative, and palliative services across the life course. Although conceptualizations of PHC vary, the 2018 Declaration of Astana reaffirmed its role as “the most inclusive, effective, and efficient approach to enhance people’s physical and mental health and well-being”^A^ and as a critical pathway toward Universal Health Coverage (UHC) and the health-related SDGs by 2030.^1^

Brazil and China illustrate two different routes to PHC strengthening. Brazil shows the value of community trust, territorial responsibility, and locally embedded teams. China shows the capacity of state-led investment and digital infrastructure to scale services rapidly. Their comparison is useful because each model compensates for a weakness in the other: Brazil's relational model needs more stable financing and integration, while China's model needs stronger trust and frontline credibility. This narrative commentary draws on selected empirical studies, policy documents, and recent WHO and national reports on PHC reforms in Brazil and China since 2015–2026.

### Developments of PHC in Brazil and its challenges

Brazil’s FHS, launched in 1994 under the Unified Health System (SUS) and adopted as a national strategy in 2006, is among the most ambitious PHC expansions in the Global South. Through multidisciplinary teams with specialist “matrix support”, FHS emphasizes proactive outreach, longitudinal relationships, and territorial accountability. By 2023, it covered over 70% of Brazil’s population, particularly benefiting underserved rural and urban peripheries. Evidence links FHS expansion to improved access, fewer preventable hospitalizations, lower mortality (including among children and older adults), and greater equity and efficiency, with resilience value highlighted during COVID−19, when higher FHS coverage was associated with lower death rates and higher vaccination uptake.^2^, ^3^, ^4^

Central to Brazil’s success is its integration of community health agents. These locally recruited individuals serve to bridge formal health services and households. Through regular home visits, health education, immunization outreach, and facilitation of early risk detection, they embody the core principles of primary healthcare including first-contact accessibility, continuity, comprehensiveness, and coordination.^5^ Community health agents strengthen disease prevention and vaccine delivery while leveraging trusted community relationships to boost residents’ confidence and uptake. The FHS aligns closely with SUS’s constitutional mandate of universality and equity, embedding PHC within a rights-based framework, a model particularly effective in reaching marginalized populations.

Brazil’s digital transformation of PHC has consolidated around e-SUS APS over the past decade, integrating the Electronic Citizen’s Medical Record and other modules to standardize records, monitor indicators, and support management. Earlier official-data analyses found that, by 2019, 92.2% of Brazilian municipalities had reached at least some degree of e-SUS AB implementation.^6^ The 2023 creation of the Secretariat for Information and Digital Health and the development of the National Health Data Network (RNDS) have advanced interoperability between national health information systems, although continued investment in workforce training is needed to improve record quality.

Despite these strengths, the FHS faces mounting pressures. Chronic underfunding has constrained operational capacity and infrastructure upgrades. Turnover among health professionals remains high due to precarious working conditions and limited career progression. Political shifts have periodically weakened federal support, leading to uneven implementation across states. While the FHS excels in preventive and basic curative care, its integration with secondary and tertiary levels remains fragmented, limiting its capacity to manage complex chronic conditions and creating delays for patients requiring specialized care.

### Developments of PHC in China and its challenges

China, by contrast, has adopted a distinctly technology-driven and centrally coordinated strategy in its PHC reforms. Since 2009, the Chinese government has prioritized PHC in its health system reforms, increasing financial investment, expanding infrastructure, and promoting universal health insurance coverage. Historically dominated by hospital-centric care, the system now explicitly promotes a “PHC-first” model, encouraging patients to seek care at township hospitals and urban community centers before accessing higher-level facilities.

According to national statistics, basic medical insurance covered approximately 1.326 billion people (around 95%) at end−2024.^B^ Family-doctor contracted services have continued to expand, prioritizing older adults and people with chronic conditions. The scope of basic public health services has been broadened with stronger emphasis on chronic disease management and integration of public health and clinical care, increasingly supported by digital tools such as interoperable electronic records, telehealth, and platform-enabled follow-up and referrals.^7^ China has also promoted county-level integrated medical and health consortia to improve coordination between PHC institutions and higher-level hospitals.

The speed and scale of digital transformation in PHC stands out. National initiatives such as “Internet Plus Healthcare” have rapidly expanded telemedicine, electronic health records, mobile-health applications, and AI-enabled tools are being promoted in some routine services. By end−2022, the national telemedicine collaboration network exceeded 95% of Chinese counties and districts.^8^ A systematic review of China’s comprehensive PHC reforms found improved service volume and chronic-disease management indicators at primary facilities, although evidence remains mixed on first-contact utilization and quality-of-care processes.^7^

Yet, despite increased access, the practical use of PHC for first-contact treatment remains limited in many settings. Many patients bypass PHC for hospitals due to concerns about provider skills, fragmented care, and lack of trust, reinforcing a perception that PHC is mainly for immunization and prescription refills rather than reliable first-contact treatment.^9^ Shortages of well-trained PHC professionals persist in rural regions, alongside high turnover and insufficient incentives. Poor integration between PHC and hospitals, fragmented financing, and weak coordinated governance further hinder efficiency. Moreover, high input-based government investment may inadvertently reduce the incentive for township health centers to improve service delivery, creating dependency rather than performance-driven improvement.^9^

### A comparative framework: four dimensions of sustainable PHC

The Brazilian and Chinese experiences can be integrated along four complementary dimensions of sustainable PHC.

***Community embeddedness.*** Brazil’s FHS shows how locally recruited community health agents, through home visits, health education, risk detection, and vaccination outreach, build durable relationships that increase residents’ confidence and uptake. China could adapt this by mobilizing volunteer non-professional agents (such as healthy retirees) to support communication, reminders, and digital navigation for older and hard-to-reach groups.

***Financial protection and sustainability.*** Brazil’s chronic underfunding and China’s risk of “investment dependency” point to the same lesson: PHC funding must be predictable and protected, yet also strategically purchased and tied to measurable quality, equity, and outcomes. Therefore, facilities need to strengthen clinical competence, continuity, and prevention performance rather than merely expanding inputs, infrastructure, or reporting activity.

***Continuity of care and coordination across levels.*** Integration across levels is the real test of PHC sustainability. Prioritizing cost-effective services, including prevention, immunization, and chronic disease management, works best when teams, agents, and information systems operate as one, and when referral pathways and shared care plans allow complex cases to move smoothly to hospitals and back. Without this, trust gains and quality improvements will be undermined by fragmented care and bypassing.

***Digital support and its dual equity effect.*** Interoperable records, telehealth, and decision-support tools can standardize workflows, support risk stratification, and improve follow-up at scale.^7^ The aim is not to replace clinicians but to reduce unwarranted variation, shorten learning curves, and make continuity and prevention performance visible and manageable. China’s experience reveals a dual equity pattern. Telemedicine has helped narrow the rural-urban gap by extending higher-level expertise into county and township facilities, with rural residents reporting improved perceived accessibility, particularly within compact medical communities and family-doctor systems.^8^ Yet the gap between older and younger users has widened, with the digital usage divide among older adults narrowing more slowly than the access divide.^10^ Without offline pathways, age-friendly interfaces, and human-mediated digital navigation, digital PHC can be equity-enhancing across geography but equity-eroding across age.

## Implications for other LMICs

Brazil and China are large, upper-middle-income countries with substantial fiscal space, while many LMICs face tighter constraints. For LMICs with constrained fiscal and workforce capacity, the policy priority should be sequenced rather than technology led. The first step is to strengthen low-cost, community-facing PHC infrastructure through community health workers, regular home visits, vaccination outreach, and simple chronic disease registries that enable frontline teams to identify, follow up, and support high-risk patients. Digital tools should then be introduced to reinforce continuity of care, rather than replace trusted frontline providers. Basic electronic registries, mobile-phone follow- up, and shared patient records may be more feasible and equitable than expensive AI systems or advanced telemedicine platforms.

## Conclusions

Brazil and China offer two converging paths toward PHC sustainability. Brazil reminds us that health begins in the home and the neighborhood, nurtured by human connection. China illustrates how innovation can extend reach and efficiency. The future of health depends on synthesizing these strengths, building systems that are not only accessible and affordable but also competent, compassionate, and resilient. In an era of pandemics, climate change, and widening inequality, such systems are essential, not optional.

## Abbreviations

PHC, Primary Health Care; UHC, Universal Health Coverage; SDG, Sustainable Development Goals; FHS, Family Health Strategy; SUS, Sistema Único de Saúde (Brazil’s Unified Health System); e-SUS APS, e-SUS Atenção Primária à Saúde; RNDS, Rede Nacional de Dados em Saúde; LMICs, Low- and Middle-Income Countries

## CRediT authorship contribution statement

**Jian Wang:** Supervision, Conceptualization. **Ivana Barreto:** Writing – review & editing, Supervision. **Garibaldi Gurgel:** Writing – review & editing. **Luiz Andrade:** Writing – review & editing. **Jialong Tan:** Writing – review & editing, Writing – original draft, Conceptualization. **Chen Chen:** Writing – review & editing, Writing – original draft, Conceptualization.

## Ethics approval and consent to participate

Not applicable.

## Consent for publication

Not applicable.

## Funding

This research was funded by 10.13039/501100001809National Natural Science Foundation of China (No. 72304214 and No. w2523045). NSFC had no role in the design, execution, interpretation, or writing of the manuscript.

## Declaration of Generative AI and AI-assisted technologies in the writing process

During the revision of this manuscript, the authors used AI-assisted language support to improve clarity, structure, grammar, and formatting. The authors reviewed and edited all AI-assisted outputs, verified the accuracy of the content, and take full responsibility for the final manuscript.

## Declaration of Competing Interest

The authors declare no competing interests.

## Footnotes

^A^ https://www.who.int/publications/i/item/WHO-HIS-SDS−2018.61

B National Healthcare Security Administration of China. 2024 Medical Insurance Development Statistical Bulletin. Beijing: NHSA; 2025. Available from: http://www.nhsa.gov.cn/

## References

[1] United Nations General Assembly. Transforming our world: the 2030 Agenda for Sustainable Development. Resolution A/RES/70/1. New York: United Nations; 2015.

[2] Macinko J., Harris M.J. (2015). Brazil’s family health strategy delivering community-based primary care in a universal health system. N Engl J Med.

[3] Giovanella L., Bousquat A., Schenkman S. (2021). The Family Health Strategy coverage in Brazil: what reveal the 2013 and 2019 National Health Surveys. Cien Saude Colet.

[4] Kessler M., Thumé E., Marmot M. (2021). Family Health Strategy, Primary Health Care, and Social Inequalities in Mortality Among Older Adults in Bagé, Southern Brazil. Am J Public Health.

[5] Martins F.P., Vigurs C., Veras M.M. (2025). Infrastructure-health nexus in Brazil: a scoping review. Glob Health Res Policy.

[6] Coelho Neto G.C., Andreazza R., Chioro A. (2021). Integration among national health information systems in Brazil: the case of e-SUS Primary Care. Rev Saude Publica.

[7] Cai C., Xiong S., Millett C., Xu J., Tian M., Hone T. (2023). Health and health system impacts of China’s comprehensive primary healthcare reforms: a systematic review. Health Policy Plan.

[8] Huang Z., Wan X., Zhou S., Yu M. (2025). Telemedicine in Action: Improving Perceived Healthcare Accessibility in Rural China. Health Care Sci.

[9] Li X., Krumholz H.M., Yip W. (2020). Quality of primary health care in China: challenges and recommendations. Lancet.

[10] Li S., Ouyang Y., Hu M. (2025). Ten-year trends of the digital divides and its effect on healthy aging among older adults in China from 2011 to 2020. NPJ Digit Med.

